# Repeatability and Validity of Different Methods to Determine the Anaerobic Threshold Through the Maximal Multistage Test in Male Cyclists and Triathletes

**DOI:** 10.3390/jfmk10010009

**Published:** 2024-12-27

**Authors:** Iñaki Llodio, Javier Yanci, Mikel Usandizaga, Auritz Larrea, Aitor Iturricastillo, Jesús Cámara, Cristina Granados

**Affiliations:** 1 AKTIBOki, Research Group in Physical Activity, Physical Exercise and Sport, Department of Physical Education and Sport, Faculty of Education and Sport, University of the Basque Country (UPV/EHU), 01007 Vitoria-Gasteiz, Spain; javier.yanci@ehu.eus (J.Y.); aitor.iturricastillo@ehu.eus (A.I.);; 2 Society, Sports and Physical Exercise Research Group (GIKAFIT), Department of Physical Education and Sport, Faculty of Education and Sport, University of the Basque Country (UPV/EHU), 01007 Vitoria-Gasteiz, Spain; 3 Physical Activity, Exercise, and Health Group, Bioaraba Health Research Institute, Basque Country, 01007 Vitoria-Gasteiz, Spain; 4 Faculty of Education and Sport, University of the Basque Country (UPV/EHU), 01007 Vitoria-Gasteiz, Spain

**Keywords:** lactate threshold, aerobic capacity, endurance performance, endurance assessment, exercise testing

## Abstract

**Objectives**: The aim of this study was to analyze the repeatability and validity of different methods to determine the anaerobic threshold through a maximal multistage cycling test; **Methods**: In total, 17 male endurance-trained athletes [7 cyclists and 10 triathletes, age 33.2 ± 6.9 yr, workload at maximal lactate steady state (MLSS_W_) 268 ± 27 W] participated in the study. The participants performed a maximal multistage cycling test twice to analyze the repeatability of the anaerobic threshold (AT) using nine different methods. In the remaining sessions, several 20 min constant load tests were performed to determine MLSS_W_ (gold standard); **Results**: The workload corresponding to 73% of the maximal power (AT_73Pmax_) showed the best repeatability followed by the Dmax method calculated from the blood lactate concentration ([La^−^]) associated with the Minimum Lactate Equivalent and final [La^−^] (Dmax_LE_). Validity analyses showed that all AT determined in the present study were strong predictors of MLSS_W_, however, AT_73Pmax_ and the workloads at 1.5 mmol·L^−1^ above the first lactate threshold significantly underestimated MLSS_W_. The use of correction equations for these variables lowered their absolute mean bias to <5 W. Dmax_LE_ and workload associated with the 86% of the maximal heart rate showed the narrowest limits of agreement to estimate MLSS_W_ closely followed by corrected AT_73Pmax_; **Conclusions**: AT_73Pmax_, using the correction equation and Dmax_LE_, stand out as powerful predictors of MLSS_W_ among the variables analyzed in the present study in trained cyclists or triathletes. Sports physiologists and coaches can use corrected AT_73Pmax_ and Dmax_LE_ to accurately assess athletes’ endurance capacity and prescribe their training.

## 1. Introduction

Endurance-cycling and triathlon are sports in which the aerobic capacity of athletes is crucial for their competitive performance [1,2]. The exercise intensity corresponding to the maximal lactate steady state (MLSS_W_), the highest workload that can be maintained over time without continual blood lactate accumulation [3,4], is regarded by many sport and exercise physiologists as one of the reference methods for the assessment of aerobic capacity [5,6,7,8,9,10]. MLSS_W_ estimates performance in endurance sports lasting 30–60 min [4,11,12]; it is unique to each individual and it can be useful to establish training intensities [13] since authors have presented evidence that exercising at workloads where blood lactate concentration remains stable may reduce risk of overtraining [14]. In addition, MLSS_W_ defines the genuine boundary discriminating between the heavy and severe exercise intensity domains [15,16]. These studies accentuate the importance of MLSS_W_ identification in the physiological profiling of endurance cyclists or triathletes because establishing training intensities based on the MLSS_W_ concept avoids overtraining and reduces the risk of injury [16,17].

The determination of the MLSS_W_ requires the performance of 3–6 subsequent tests on separate days. As this procedure is time-consuming and may interfere with the training process, simpler methods based on a single maximal multistage test measuring ventilation or blood lactate concentration ([La^−^]) are usually utilized to estimate MLSS_W_. The exercise intensities determined based on these methods are usually referred to as anaerobic threshold. Equipment for measuring gas exchange is quite expensive, so it is interesting to look for more cost-effective alternatives. Among the methods that require [La^−^] measurement, estimators based on the first lactate threshold [18,19,20,21], fixed [La^−^] [11,18,20], and the modified Dmax method [22,23] are commonly utilized as surrogates for estimating MLSS_W_. Note that some predictors of MLSS_W_ are based on the first lactate threshold, even though this threshold represents a lower exercise intensity than MLSS_W_. Maximal aerobic workout (Pmax) or fixed percentages of Pmax and maximal heart rate (HRmax) have also been proposed as MLSS_W_ predictors [6,7,18]. However, the generalizability of these MLSS_W_ estimates is limited to the running protocols previously employed. It is, therefore, uncertain whether these MLSS_W_ estimates apply to other exercise modes, such as cycling.

The assessment of repeatability is an important aspect of examining alternative methods because measurement errors can seriously affect statistical analysis and interpretation. Determining minimum detectable changes (MDC) of MLSS_W_ predictors is especially useful because it allows exercise physiologists, trainers, and sports practitioners to distinguish individual actual changes in anaerobic thresholds from the measurement error. As far as we are concerned, the repeatability, MDC, and validity of most of the MLSS_W_ predictors abovementioned have not been compared. Therefore, the aims of this study were to compare the repeatability, MDC, and validity (considering the MLSS_W_ as the gold standard) of different cost-effective methods to determine the anaerobic threshold through a maximal multistage test in male cyclists and triathletes. This study focused on methods based on the Minimum Lactate Equivalent (LEmin, the minimum value of the [La^−^]/Workload vs. Workload curve fitting during an incremental test) related intensities, workload at fixed [La^−^], Dmax, and fixed relative HR and power values.

## 2. Materials and Methods

### 2.1. Participants

Seventeen amateur male endurance-trained athletes who were at the level of tier 2 [23] (seven cyclists and ten triathletes; means ± standard deviation, age 33.2 ± 6.9 yr, height 177.7 ± 8.3 cm, body mass 74.9 ± 10.1 kg, body fat 8.6 ± 2.9%) voluntarily participated in this study. Participants were required to meet the following inclusion criteria: (1) being a male triathlete or cyclist aged between 18 and 45 years, (2) had been consistently involved in triathlon or cycling training for at least 2 years and had participated in a triathlon or cycling event prior to participation in the study, and (3) had a training routine of ≥2 cycling training sessions a week during the last two months. Some participants were triathletes and, in addition to ≥2 bicycle sessions, also trained in running and swimming. All participants completed at least four endurance sessions per week. The exclusion criteria were (1) taking any medications or substances known to influence physical performance, [La^−^] or HR, and (2) having any musculoskeletal injury and illness that might limit their full participation. Written informed consent was obtained from all participants before the start of this study, which was approved by an Ethics Committee (code M10_2018_215) and conformed to the guidelines of the Declaration of Helsinki [24].

### 2.2. Study Design

A repeatability and validity study was conducted to determine MLSS_W_ from a maximal multistage cycling test (MMCT). For each participant, testing was conducted over 8–11 laboratory sessions separated by at least 2 days. In the first session, an initial evaluation (a training history and injury and illness questionnaire), anthropometric measurements, and a preliminary familiarization cycling trial were performed. In the second and third sessions, participants performed an identical maximal multistage cycling test (MMCT_1_ and MMCT_2_) to calculate the anaerobic threshold or its estimator (AT) using nine different methods and their repeatability. In the remaining sessions, several 20-min constant load tests (CLTs) were performed to determine twice MLSS_W_ for each participant. The mean value of two MLSS_W_ determinations as a proxy of aerobic cycling performance was used to study the validity of different ATs.

### 2.3. Testing Procedures

The same researcher took measurements on every cyclist and triathlete at a similar time of day (±2.5 h) for each of them. All participants were required not to engage in any vigorous activity during the 2 days before each test. To facilitate the replenishment of carbohydrate stores and adequate hydration status during the period of the study, they received specific instructions on how to increase their dietary carbohydrate intake and hydration. The athletes were required to maintain their training protocol in terms of volume and intensity, and their lifestyle was unchanged (i.e., physical activity and hours of sleep). They were also required to have abstained from alcohol and caffeine intake for 12 h and from a substantial meal for 2 h. All the cycling trials were performed in the same facility and on the same cycloergometer (ERGelek EG2, Vitoria-Gasteiz, Spain), which was adapted for use with triathlon bars and calibrated by the manufacturer prior to the experiment. The cycloergometer provided work rates that were independent of pedal frequencies. The total test period for a participant was shorter than 8 weeks. The athletes used the same cycling shoes, and no fan was used for the tests. The room temperature during the whole testing period was 18.6 ± 2.3 °C and the relative humidity (49 ± 9%).

### 2.4. Preliminary Trial

During the preliminary trial, participants were familiarized with the methodology after they had made adjustments to the cycloergometer for proper fit. This trial consisted of a submaximal multistage exercise in a cycloergometer up to 85% of the estimated HRmax (HRmax = 220 − age). The athletes were free to choose any constant pedal cadence between 75 and 95 rpm and were required to maintain the exact cadence chosen in the following tests. No data from the preliminary trial were analyzed.

### 2.5. Maximal Multistage Cycling Test (MMCT)

A MMCT was conducted twice, 2–6 days apart, to determine duplicate AT. The test started at 30 W and increased by 30 W every 3 min until volitional exhaustion. This protocol, which was similar to that used in other studies with trained cyclists [25,26,27,28,29], was chosen to ensure that an adequate number of blood [La^−^] measuring points were used both as the basis lactate and for the determination of the LEmin [18]. Stage duration of 3 min was chosen because it is the minimum stage duration needed [La^−^] to remain stable in each exercise stage [20]. The test terminated when the participants could not maintain the pedal cadence despite strong verbal encouragement. Perceived exertion was determined at the end of each MMCT [30]. Maximal effort criteria were considered to verify the outcomes, from which participants must have reached at least two from the list: (a) failure of HR to increase with further increases in exercise intensity, (b) [La^−^] > 9 mmol·L^−1^, and (c) a rating of perceived exertion (RPE) ≥ 18 on the 6–20 scale. Blood samples were collected while the participant was cycling to measure [La^−^] at rest, at the last 15 s of each stage, and at the end of the test. An earlobe was cleaned and dried before puncturing by a lancet for blood sampling. After discarding the first drop, a capillary blood sample was obtained and [La^−^] was determined via amperometric measurement using a portable analyzer (Lactate Pro 2; Arkray, Kyoto, Japan). Participants were blinded to the elapsed time, exercise intensity, and physiological measurements but they continuously received visual feedback for the pedal cadence. The maximal aerobic workout (Pmax) was estimated as follows: Pmax = Power of last completed stage (W) + [t (s)/180·30] where ‘t’ is the time sustained during the incomplete stage. HR was registered at 1 s intervals using an HR monitor (Sportester, Polar, Kempele, Finland) and HRmax was considered as the highest 5 s average.

Five ATs and four estimators of the theoretical AT were determined mathematically during MMCT. The ATs determined mathematically during MMCT were the following: (1) the workload at 1.5 mmol·L^−1^ above the [La^−^] associated with the lowest stage above which [La^−^] increased by ≥0.1 mmol·L^−1^ in the following stage and ≥0.2 mmol·L^−1^ in the subsequent stage (LT1_+1.5mM_) [18]. (2) The workload corresponding to the Minimum Lactate Equivalent (LEmin) plus 1.5 mmol·L^−1^ (LE_+1.5mM_). The workload at 1.5 mmol·L^−1^ above the [La^−^] associated with the LEmin in the individual [La^−^] vs. workload second-order polynomial curves. The LEmin was considered the minimum value of the quotient [La^−^]/Workload in the individual [La^−^]/Workload vs. Workload second-order polynomial curves. Using an appropriate protocol, the incremental test produces a “U-shaped” curve. The lower limit of this curve is considered the intensity at the LEmin. The [La^−^] that corresponded to this intensity was determined in the individual [La^−^] vs. workload second-order polynomial curves. Once 1.5 mmol·L^−1^ was added to the determined [La^−^], the intensity corresponding to the resulting [La^−^] was determined in the last mentioned curves. For more details, see Berg et al. [31]. (3) The workload at 1.5 mmol·L^−1^ in the individual [La^−^] vs. workload second-order polynomial curves above the average of the first four [La^−^] values of exercise (LT2) [32]. (4) The workload at the maximum perpendicular distance from the straight line between the [La^−^] is associated with the Minimum Lactate Equivalent and final [La^−^] data point in the third-order polynomial curve describing the [La^−^] kinetics during the MMCT (Dmax_LE_) [22]. (5) The workload at the maximum perpendicular distance from the straight line between the [La^−^] associated with the previous stage to the one that the [La^−^] increased ≥0.4 mmol·L^−1^ and final [La^−^] data point in the third-order polynomial curve describing the [La^−^] kinetics during the MMCT (Dmax_0.4_) [21]. The four estimators of the theoretical AT determined mathematically during MMCT were the following: (1) and (2) the workloads at fixed [La^−^] of 3.5 mmol·L^−1^ (LT_3.5mM_) and 4 mmol·L^−1^ in the individual [La^−^] vs. workload second-order polynomial curves (LT_4mM_) [20]. (3) The workload corresponds to 73% of the Pmax (AT_73Pmax_). The specific value of 73% was chosen because the average taken across a series of studies using different participant characteristics and protocols showed that cycling the MLSS_W_ corresponded, on average, to 73% of the maximal workload (Pmax) [33,34,35]. (4) The workload at 86% of HRmax (AT_86HRmax_). The specific value of 86% was chosen to take into account the study by Snyder et al. [36], who showed that this is approximately the percentage of HRmax associated with the intensity of MLSS_W_. HR was plotted against workload, and a second-degree polynomial regression fit was calculated. The resulting formula was used to determine AT_86HRmax_. Note that the power values corresponding to AT are only expressed in absolute units for clarity. Coefficients of determination (R^2^) of the individual second-order [La^−^] vs. workload curves were all >0.85, of the third-order [La^−^] vs. workload curves and of the HR vs. workload curves, they were all >0.97; of the individual [La^−^]/Workload quotient vs. workload, second-order polynomial curves were all >0.71.

### 2.6. Constant Load Tests for the Determination of MLSS_W_

Athletes completed five to eight 20-min CLTs at different workloads to determine and duplicate the exercise intensity corresponding to the maximal lactate steady state (MLSS_W1_; MLSS_W2_). Blood samples were taken at rest and at the 10th and 20th min of exercise with the same equipment and procedure used in the MMCTs. The workload of the first CLTs corresponded to 73% of the Pmax reached during the second MMCT. The workload of the first CLT of the MLSS_W2_ corresponded to MLSS_W1_. If, during this first CLT, a steady state or a decrease in [La^−^] was found, the workload was increased by 20 W, and subsequent CLTs were performed until no steady state of [La^−^] was observed. Conversely, if the first CLT resulted in a clearly identifiable increase in [La^−^], subsequent CLTs were performed at 20 W lower velocities until a steady state [La^−^] was reached. The process of increasing or decreasing workload by 20 W, or later by 10 W, in subsequent tests was further repeated until MLSS_W_ was determined twice to a precision of 10 W. Thus, each participant had to perform at least five CLTs to determine two MLSS_W_-s. The CLTs were separated by at least 2 days. An increase of ≤0.5 mmol·L^−1^ in [La^−^] during the final 10 min of exercise (0.05 mmol·L^−1^·min^−1^) was defined as the criterion for [La^−^] to be considered at a steady state [6]. The MLSS_W_ was defined as the highest workload meeting this stability criterion. In most studies, MLSS_W_ was determined during a CLT lasting 30 min, and a [La^−^] increase ≤ 1.0 mmol·L^−1^ (0.05 mmol·L^−1^·min^−1^) between the 10th and the 30th min of exercise set as the stability criteria. In the present study, however, because of time limitations, MLSS_W_ was determined by analyzing the change in [La^−^] between the 10th and the 20th min of CLTs, and MLSS_W_ was also defined as an increase ≤ 0.05 mmol·L^−1^·min^−1^ in the 10 last minutes of exercise. Using the mentioned lactate stability criteria, CLTs lasting only 20 min can be adequate for MLSS_W_ determination since no difference in the MLSS_W_ was found when 20 min or 30 min CLTs were used [37]. HR was averaged every 30 s of exercise. The average value of HR measured at 10, 15, and 20 min of exercise was considered the mean HR value at MLSS_W_. The average value of [La^−^] measured at 10 and 20 min of exercise was considered the mean lactate value at MLSS_W_.

### 2.7. Statistical Analyses

Standard statistics were used to calculate means and standard deviations (SD). Normal data distribution was analyzed using the Shapiro–Wilk test. The Paired Student’s *t*-test and the Wilcoxon test were used, respectively, for normally and not normally distributed data (work rate at Dmax_0.4_) to compare MMCT_1_ and MMCT_2_. The magnitudes of the differences were assessed using 90% confidence intervals (CI) and Hedges’ g effect sizes (ES). Repeatability of ATs was analyzed, comparing the consistency between trials (i.e., MMCT_1_ vs. MMCT_2_) by calculating the mean of the intra-subject SD (the SDs were log-transformed before calculating the average, and then back-transformed using an exponential function) and the mean of the untransformed intra-subject coefficient of variation (CV), by the standard error of measurement (SEM) calculated from the two way Analysis of Variance [38], by the intraclass correlation coefficient (ICC, two-way mixed effects, absolute agreement and single measurement model) as suggested by Koo et al. [39] and by the 95% limits of agreement method (LoA; mean difference ± 1.96 SD) [40]. Friedman’s test, with pairwise comparison post-hoc tests, was used to compare intra-subject CV and intra-subject SD between ATs. Pearson’s correlation coefficients (r) were used to determine the association between the mean of ATs and their repeatability in terms of intra-subject SD and intra-subject CV. The r values of 0.1–0.3, 0.3–0.5, 0.5–0.7, 0.7–0.9, and ≥0.9 were considered to represent *small*, *moderate*, *large*, *very large*, and *nearly perfect* associations, respectively [41]. The MDC was calculated as follows: MDC = SEM × 1.96 × √2 [38].

Agreement with the reference method (MLSS_W_) was assessed over the mean values by the standard error of the estimate (SEE) and by the Bland–Altman method. When the absolute mean difference, i.e., the absolute mean of the bias, was higher than 6 W, simple regression analyses were applied to produce correction equations. The corresponding AT was used as the independent variable and MLSS_W_ was employed as a dependent variable in the stepwise regression analyses. Pearson’s correlation coefficients were performed to assess associations between MLSS_W_ and workload at AT measured at MMCT. Correlation magnitudes were interpreted as described above. Statistical significance was set at *p* ≤ 0.05. Statistical analyses were conducted using IBM SPSS Statistics 27.0 (SPSS Inc., Chicago, IL, USA).

## 3. Results

### 3.1. Repeatability of Anaerobic Thresholds Through MMCTs

All the participants completed both MMCTs, and all ATs could be calculated in all participants. Mean Pmax, HRmax, final [La^−^], and final RPE at MMCT_1_ (349 ± 34 W, 175 ± 8 beats·min^−1^, 10.9 ± 3.7 mmol·L^−1^, 19.3 ± 0.9 UA) were not significantly different from MMCT_2_ (351 ± 38 W, 176 ± 8 beats·min^−1^, 10.8 ± 3.3 mmol·L^−1^, 19.4 ± 0.9 UA). Table 1 shows descriptive characteristics and the repeatability results of ATs at MMCT_1_ and MMCT_2_. There were no significant differences in the analyzed variables between MMCTs except for the workload at Dmax_LE_, which was lower at MMCT_1_ than at MMCT_2_. All ATs showed *nearly perfect* ICC except LT_4mM_, which showed a *very large* value. AT_73Pmax_ showed the best repeatability results regarding intra-subject SD, intra-subject CV, Bland–Altman LoA, SEM, and MDC. When ATs were expressed as HR, very large ICC values were found except for AT_86HRmax_ and AT_73Pmax_, which showed *nearly perfect* values. Heart rate at AT_86HRmax_ showed the best repeatability values in terms of, intra-subject CV, Bland–Altman LoA, SEM, and MDC.

**Figure 1:**
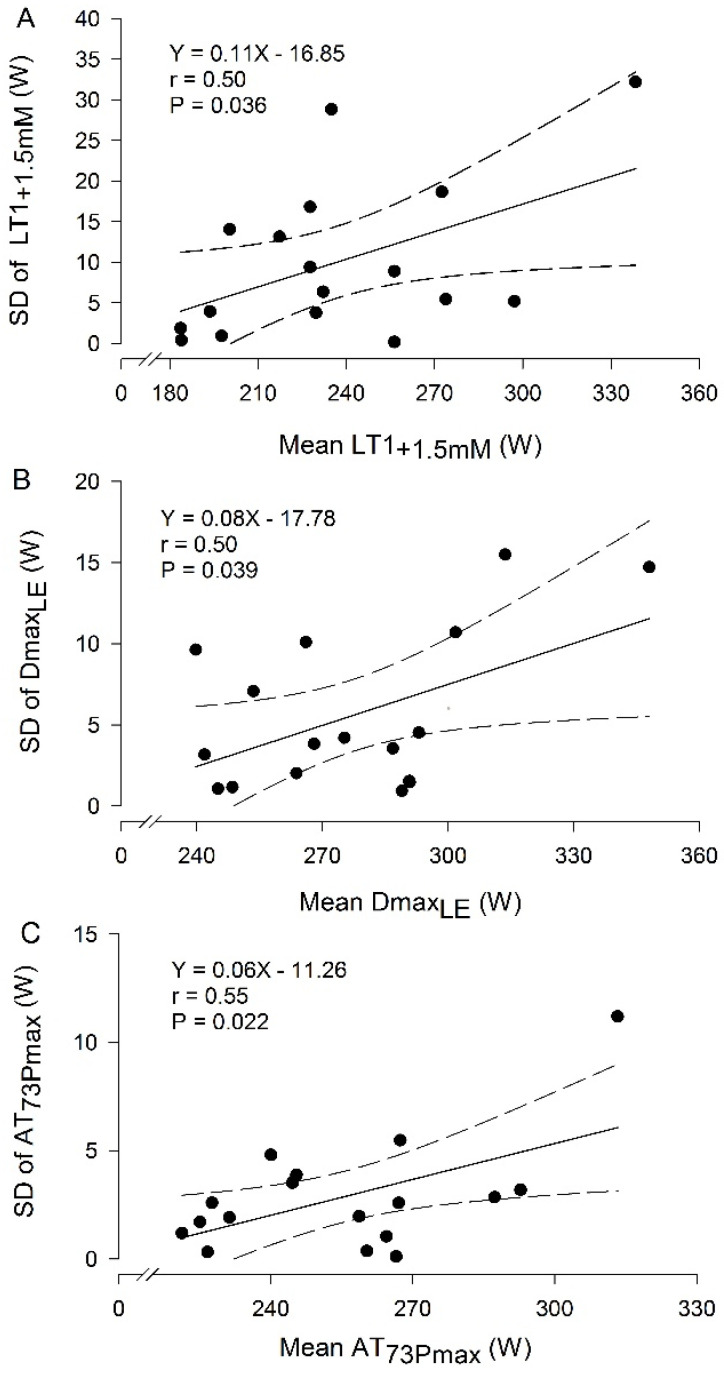
Linear relationship between the workload at (**A**) LT1_+1.5mM_, the workload at 1.5 mmol·L^−1^ above the [La^−^] associated with the lowest stage above, which [La^−^] increased by ≥0.1 mmol·L^−1^ in the following stage and ≥0.2 mmol·L^−1^ in the subsequent stage, (**B**) Dmax_LE_, workload at the maximum perpendicular distance from the straight line between the [La^−^] associated with the Minimum Lactate Equivalent and final [La^−^], and (**C**) AT_73Pmax_, workload at 73% of Pmax, and their respective intra-subject SD. Solid lines represent linear regression and dashed lines represent 95% confidence intervals.

The association between the mean (MMCT_1_ and MMCT_2_) ATs and their repeatability in terms of intra-subject SD and intra-subject CV was analyzed to see if the measurement variation was homogeneous throughout the range of mean AT values. LT1_+1.5mM_, Dmax_LE_, and AT_73Pmax_ expressed as workloads and their respective intra-subject SD values were the only variables that showed statistically significant associations (Figure 1A, Figure 1B, and Figure 1C, respectively).

### 3.2. Constant Load Tests

One participant did not complete any of the CLTs due to an illness; therefore, 16 participants completed all the CLTs required to determine twice the MLSS_W_. The mean of the individual MLSS_W_ values was 268 ± 27 W (289 ± 25 W in cyclists and 256 ± 21 W in triathletes).

### 3.3. Validity of Anaerobic Thresholds

The validity analysis was performed by comparing the workloads at ATs against MLSS_W_ (Table 2). The mean workloads corresponding to AT_73Pmax_, LT2, LE_+1.5mM_, and LT1_+1.5mM_ were significantly lower than MLSS_W_. All ATs showed *nearly perfect* associations with MLSS_W_ except LT1_+1.5mM_, which showed a *very large* association (Table 2). Dmax_LE_, AT_73Pmax_, and AT_86HRmax_ showed the narrowest LoA. The absolute mean bias for LT1_+1.5mM_, LE_+1.5mM_, LT2, LT_3.5mM_, and AT_73Pmax_ was higher than 6 W; consequently, correction equations were performed for the mentioned ATs (Table 3). The use of correction equations lowered the absolute mean bias to <5 W.

**Figure 2:**
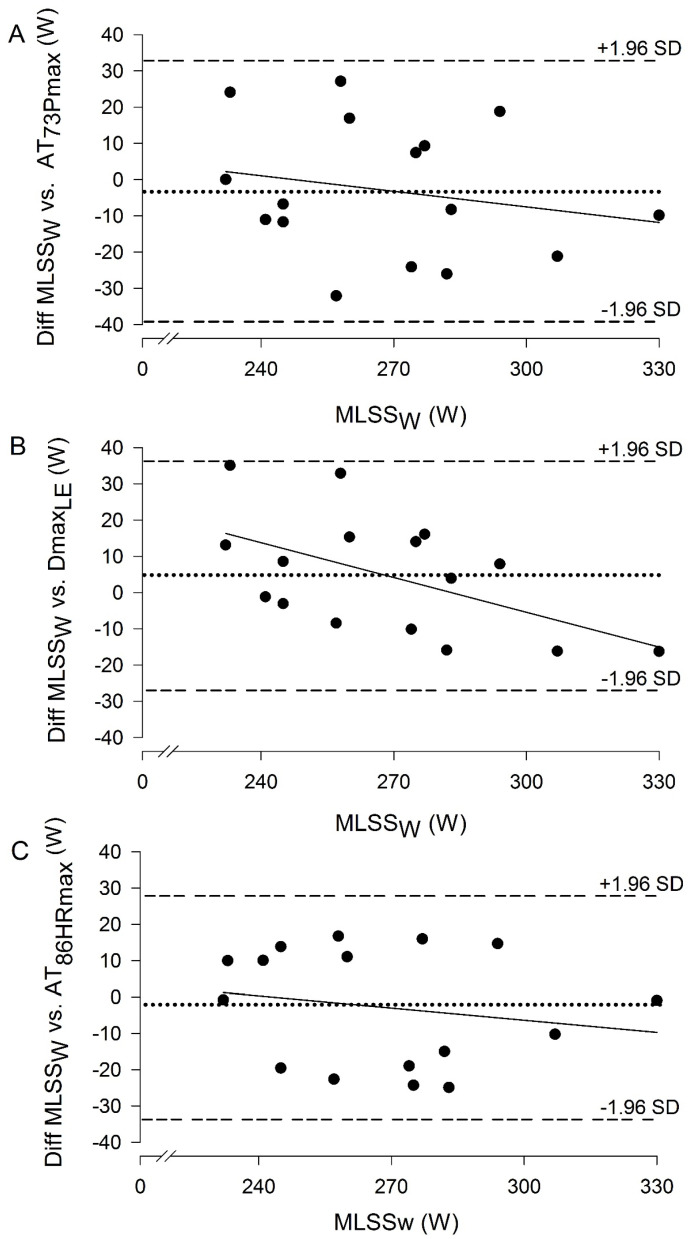
Bland–Altman plots show the difference between the workloads at anaerobic thresholds over the MLSS_W_. Anaerobic thresholds correspond to the following. (**A**) Corrected AT_73Pmax_, workload at 73% of Pmax, (**B**) Dmax_LE_, workload at the maximum perpendicular distance from the straight line between the [La^−^] associated with the Minimum Lactate Equivalent and final [La^−^], and (**C**) AT_86HRmax_, workload at 86% of maximal heart rate. The dotted horizontal lines represent the bias between the 2 measurements. The dashed horizontal lines represent the 95% limits of agreement between the 2 variables and the solid lines correspond to the regression lines.

The Bland–Altman plots of Figure 2 show the difference between the workload of corrected AT_73Pmax_, Dmax_LE_, and AT_86HRmax_ and the actual MLSS_W_ against the MLSS_W_. These plots indicate good agreement between the estimated and actual MLSS_W_ based on the low bias and relatively narrow limits of agreement. Unlike for AT_73Pmax_ and AT_86HRmax_ (*p* = 0.43 and 0.49, respectively), the gradient of the regression line was different from zero for Dmax_LE_ (*p* = 0.03), which means that Dmax_LE_ tends to slightly overestimate MLSS_W_ for cyclists with low MLSS_W_ and slightly underestimate that for the ones with high MLSS_W_.

## 4. Discussion

The objectives of the present research were to compare the repeatability, MDC, and validity (considering the MLSS_W_ as the gold standard) of different methods to determine AT through a maximal multistage test in male cyclists and triathletes. The mean MLSS_W_ in the present study was similar to the values previously reported for trained male cyclists (264 ± 39 W [42]; 255 ± 32 W [43]). Regarding workload, AT_73Pmax_ showed the best repeatability (the highest ICC, lowest intra-subject SD, intra-subject CV, SEM, and MDC, and the narrowest LoA; Table 1). Validity analyses showed that all methods used in the present study to determine AT were strong predictors of MLSS_W_; however, AT_73Pmax_, LT2, LE_+1.5mM,_ and LT1_+1.5mM_ significantly underestimated MLSS_W_. The use of correction equations for these variables lowered their absolute mean bias to <5 W. Dmax_LE_ and AT_86HRmax_ showed the narrowest LoA to estimate MLSS_W_ closely followed by AT_73Pmax_, Dmax_0.4_, and LT2. Dmax_LE_, followed by Dmax_0.4_, showed the lowest SEE. These results indicate that AT_73Pmax_, using the correction equation [Corrected AT_73Pmax_ = −69.893 + (1.332 ∗ AT_73Pmax_)] and Dmax_LE_, are the best predictors of MLSS_W_ among the variables analyzed in the present study in cyclists or triathletes. In terms of HR, AT_86HRmax_ showed the highest ICC, the lowest SEM and MDC, and the narrowest LoA. Both AT_86HRmax_ and AT_73Pmax_ showed significantly lower intra-subject SD and intra-subject CV compared to the rest of the ATs.

The workload at AT_73Pmax_ was the most repeatable AT, showing an ICC value of 0.99, followed by Dmax_LE_, LT_3.5mM_, LE_+1.5mM_, and LT2 (ICC 0.96–0.97). This result is in line with previous research showing very high repeatability of Pmax obtained during incremental tests in cycling [41,44] and treadmill running [45]. The ICC of AT_73Pmax_ in the present study compares favorably with other ATs determined in male cyclists during incremental maximal exercise tests, such as the second ventilatory threshold (ICC 0.96, [43]; ICC 0.87, [46]), respiratory exchange ratio = 1 (ICC 0.79), workloads at 0.5–3.0 mmol·L^−1^ above baseline lactate measurements (ICC 0.87–0.89), original Dmax (ICC 0.57) [43], and ATs based on surface electromyography (ICC 0.86–0.87, [47]), and is similar to the values for the workload corresponding to a 20-min time-trial in competitive male cyclists (ICC 0.98) [48]. Differences such as homogeneity and sample size of the participants [39], test protocol characteristics, and repeatability of the instruments/equipment, as well as the exact ICC model chosen for each study, might explain the differences in ICC values between studies [49]. Indeed, research has shown that ICC values were affected by ranges and slopes of the data and differed according to the different models of ICC used [50]. The mean intersubject variability (CV) in the present study was 10.4% for AT_73Pmax_ and 10.5% for Dmax_LE_. Most of the abovementioned studies had similar or slightly higher intersubject variability (CV 10.6–15.1%), which indicates that the higher ICC values for AT_73Pmax_ and Dmax_LE_ in the present study are not due to differences in the heterogeneity of the sample. The AT_73Pmax_ may be the most reliable measurement because it is a performance-based metric rather than a physiological one, unlike other thresholds or estimators that depend on [La^−^] levels or HR percentages.

The SEMs for workloads at AT_73Pmax_ and Dmax_LE_ in the present study were 3.75 W (1.5% of the mean AT_73Pmax_) and 6.24 W (2.3% of the mean Dmax_LE_), respectively. These values compare favorably with ATs based on the second ventilatory threshold determined through a ramp exercise test in cyclists (SEM 16 W) [46]. The variation in SEM values may be due to the analytical approach used [46]. The workload at AT_73Pmax_ in the present study showed significantly better repeatability in terms of intra-subject CV (1.1%) than most of the ATs. Moreover, Dmax_LE_ showed significantly better intra-subject CV (2.0%) compared to LE_+1.5mM_ and LT1_+1.5mM_. These intra-subject CVs of AT_73Pmax_ and Dmax_LE_ are slightly lower than the value of 2.8% for both LE_+1.5mM_ and LT_3.5mM_ in the study of Hoefelmann et al., determined in male cyclists during a protocol similar to the present study [26], and this value, in turn, is slightly lower than the values for the same variables in the present study (intra-subject CV for LT_3.5mM_ 3.1% and for LE_+1.5mM_ 3.4%). Pallares et al. showed intra-subject CV values of 2.1% for the second ventilatory threshold, 3.0–3.7% for ATs derived from the lactate threshold determined as the highest workload not associated with a rise in [La^−^] above baseline, 3.7% for LT_4mM_, 6.4% for respiratory exchange ratio = 1, and 10.3% for the original Dmax, determined in male cyclists during ramp protocol tests [43]. The differences in intra-subject CVs between the present study and the one by Pallares et al. could be due to the dissimilarities in the protocols (ramp vs. stage). The present results indicate that the workload at AT_73Pmax_, followed by Dmax_LE_, is more repeatable than other ATs.

Considering the MDC of ATs is important when monitoring the progress of athletes because biological variation and measurement error may incorrectly suggest a change that is not real. Since the MDC of AT_73Pmax_ is almost half that of Dmax_LE_ and approximately three times lower than the rest of the ATs, it is suggested that the workload at AT_73Pmax_ is the most sensitive method to distinguish true changes in AT from intertrial variation and measurement error. In the present study, the power output at AT_73Pmax_ and Dmax_LE_ required to detect a change in an individual physiological profile with 95% confidence was relatively low, at 4.1% and 6.2%, respectively. Significant correlations were found in the present study between individual mean LT1_+1.5mM_, Dmax_LE_, and AT_73Pmax_ expressed as workloads and their respective intra-subject SD values. Since the MDC is affected by the intra-subject SD [38], MDC may tend to be higher the higher the level of the athlete is and lower the lower the level, for the mentioned Ats. This trend may not be observed when the MDC is expressed as a percentage.

In terms of HR, AT_86HRmax_ was the most repeatable AT in the present study (ICC 0.95; intra-subject CV 0.94%; SEM 1.8 beats·min^−1^) followed by AT_73Pmax_ (ICC 0.90; intra-subject CV 1.10%; SEM 3.7 beats·min^−1^) and Dmax_LE_ (ICC 0.85; intra-subject CV 1.7%; SEM 4.6 beats·min^−1^). The ICC value for HR at AT_86HRmax_ in the present study was similar to the ICC values shown for HR during 20 min time trials in competitive male cyclists (ICC 0.94, [48]) and higher than for the respiratory compensation point determined in cyclists during ramp exercise (ICC 0.92) [46]. Hoefelmann et al. reported a slightly better repeatability for HR at LT_3.5mM_ (ICC 0.82; intra-subject CV 2.5%) compared to the one for the same variable (ICC 0.80; intra-subject CV 3.1%) and lower than the ones for AT_86HRmax_ and AT_73Pmax_ in the present study [26]. Since the MDC of HR at AT_86HRmax_ is lower than half that of HR at AT_73Pmax_ and Dmax_LE_ and approximately three times lower than the rest of the ATs, it is suggested that AT_86HRmax_ is the most sensitive AT in terms of HR to distinguish true individual changes from measurement error and biological variation.

Controversial results have been found in the literature regarding the bias of ATs to estimate MLSS_W_. The mean workloads at Dmax_LE_, AT_86HRmax_, LT_3.5mM_, LT_4mM_, and Dmax_0.4_ were not significantly different from MLSS_W_ in the present study. This is consistent with studies showing that LT_3.5mM_ [25], Dmax_LE_, and Dmax_0.4_ were not significantly different from MLSS_W_ [22,51]. In contrast, Hauser et al. showed significantly lower values for LT_4mM_ determined in a similar test protocol to the present study (increments of 40 W every 4 min) than MLSS_W_ in male participants with different endurance levels [52], and Pallares et al. showed that in trained cyclists, the mean LT_4mM_ determined during a maximal ramp test protocol (increments of 25 W·min^−1^) significantly overestimated MLSS_W_ [43]. Differences in the protocol of the maximal test and the endurance level of the participants could explain the differences between the studies. The absolute mean bias for LT1_+1.5mM_, LE_+1.5mM_, LT2, LT_3.5mM_, and AT_73Pmax_ was higher than 6 W in the present study (mean bias between —9 W and –38 W). Specifically, the uncorrected mean LE_+1.5mM_ was 31 W lower than the mean MLSS_W_, which is in agreement with the study by Hauser et al. who showed that LE_+1.5mM_ was 37 W lower than MLSS_W_ in males with different endurance levels [52]. The use of correction equations in Table 3 lowered the absolute mean bias to <5 W without harming LoAs, which suggests that when these AT values are determined through a similar test protocol to the present study in trained cyclists or triathletes, correction equations should be used.

The MLSS_W_ corresponded to 76.8% of Pmax in the present study. Previous studies have shown that experienced endurance-trained athletes tend to attain their MLSS_W_ or ventilatory-related thresholds at a higher %Pmax compared to lower-level athletes [7,25,47]. In the present study, the association between MLSS_W_ and MLSS_W_ expressed as a percentage of Pmax almost reached statistical significance (*p* = 0.052; r = 0.49 yielding the following equation MLSS_W_ in %Pmax = 54.17 + 0.09 ∗ MLSS_W_). This suggests that an uncorrected AT_73Pmax_ could agree with MLSS_W_ for people with an MLSS_W_ of about 209 W but may tend to overestimate it in people with lower endurance capacity and underestimate it in people with higher endurance levels, as is the case in the present study. This variation should be taken into account for coaches and athletes who prescribe training intensities as percentages of Pmax. The protocol used to measure MLSS_W_ could also influence the percentage of Pmax at which MLSS_W_ is observed. In the present study, we used a CLT of 20 min whereas in most of the studies, a CLT of 30 min has been used. A CLT lasting only 20 min can, however, be adequate for MLSS_W_ determination when 0.05 mmol·L^−1^·min^−1^ criteria are used for [La^−^] stability [37]

The lowest LoA values for MLSS_W_ prediction in the present study were when Dmax_LE_ (±32 W or ±11.8%), AT_86HRmax_ (±32 W or ±11.8%), and Dmax_0.4_ (±33 W or ±12.4%), as well as the corrected and uncorrected AT_73Pmax_ (±36 W or ±13.6% and 33 W or ±12.3%, respectively) and LT2 (±35 W or ±13.2% and ±34 W or ±12.7%, respectively) were used. These LoA are slightly higher than those in the study by Grossl et al. in trained cyclists predicting MLSS_W_ from LT_3.5mM_ (±25 W or ±10.3% of their MLSS_W_, which was 247 ± 33 W, CV of MLSS_W_ 13.4%) and LE_+1.5mM_ (±23 W or ±9.5% of their MLSS_W_) through a multistage maximal protocol test with an initial workload of 105 W and 35 W increments every 3 min [53] and lower than those in the study by Pallares et al. predicting MLSS_W_ from second ventilatory threshold (±39 W or ±15.3% of their MLSS_W_, which was 255 ± 32 W [43]; CV of MLSS_W_ 12.5%), workloads at [La^−^] of 1.5, 2.0, 2.5, and 3 mM·L^−1^ above baseline measurements (±41–43 W or ±16.1–16.9% of their MLSS_W_) and LT_4mM_ (±42 W or ±16.6% of their MLSS_W_) through a maximal ramp exercise test with increments of 25 W·min^−1^. The workload associated with a respiratory exchange ratio equal to unity, RER = 1 (±89 W or ±34.9% of their MLSS_W_), and Dmax threshold determined through the two endpoints of the curve (±75 W or ±29.2%) showed substantially higher values of LoA in the study by Pallares et al. [43]. Hauser et al. also showed higher LoAs for LT_4mM_ and LE_+1.5mM_ (±49 W or ±22% of their MLSS_W_, which was 221 ± 43 W; CV of MLSS_W_ 19.5%) in males with a heterogeneous endurance level [52]. Differences in the heterogeneity of the sample (CV of MLSS_W_ in the present study was 10.1%), in the protocol of the multistage maximal tests, and in the exact method for AT determination could explain the differences in the LoAs. The effects of the multistage protocol test design and the exact methods for AT determination on the validity of MLSS_W_ prediction have been precisely highlighted by Jamnick et al. [42].

The present investigation is limited in some aspects. Many tests were conducted with each participant, with over 100 tests completed across all participants; therefore, the sample size of endurance athletes was low, although similar to previous studies [43,46,48]. The applicability of the results of the present study is limited to trained cyclists or triathletes with MLSS_W_ ranging from 232 to 330 W. Caution should be taken when generalizing these results to other populations, especially to those with significantly different MLSS_W_ values and females, or to other testing procedures and equipment. Moreover, some methods used to determine the boundary discriminating between the heavy and severe exercise intensity, such as the second ventilatory threshold and critical power have not been measured. Therefore, a direct comparison between these methods and those measured in the present study has not been possible. In addition, the participants agreed to maintain their training protocol in terms of volume and intensity and to keep their lifestyles unchanged during the testing period. We assumed they adhered to this. Despite these limitations, the results of the present study provide important and novel information about the estimation of the MLSS_W_, which is considered one of the reference methods for the assessment of endurance capacity.

## 5. Conclusions

All methods analyzed in the present study to determine AT from the MMCT showed *very large* or *nearly perfect* values of repeatability in terms of ICC. AT_73Pmax_ showed the best repeatability results in terms of intra-subject SD, intra-subject CV, Bland–Altman LoA, SEM, and MDC. When ATs were expressed as HR, AT_86HRmax_ and AT_73Pmax_ showed nearly perfect values. The heart rate at AT_86HRmax_ showed the best repeatability values in terms of intra-subject SD, intra-subject CV, Bland–Altman LoA, SEM, and MDC. All ATs showed *nearly perfect* associations with MLSS_W_ except LT1_+1.5mM_, which showed a very large association. The mean bias for LT1_+1.5mM_, LE_+1.5mM_, LT2, LT_3.5mM_, and AT_73Pmax_ were higher than ±6 W; consequently, correction equations were performed for the mentioned ATs to lower mean of the bias to <5 W. These results indicate that AT_73Pmax_, using the correction equation [Corrected AT_73Pmax_ = −69.893 + (1.332 · AT_73Pmax_)] and Dmax_LE_, are the best predictors of MLSS_W_ among the variables analyzed in the present study in cyclists or triathletes. These results are valuable and of interest to sports physiologists, coaches, and athletes to test athletes’ endurance capacity and individualize their training accurately.

## Figures and Tables

**Table 1:** Repeatability of anaerobic thresholds measured through MMCT expressed in terms of workload and HR (*n* = 17).

		Mean ± SD		Difference			ICC		Bland Altman		Intra-Subject SD	Intra-Subject CV (%)	SEM	MDC
		MMCT_1_	MMCT_2_	*p*	90% CI	ES	*p*	r	Bias	LoA				
AT_73Pmax_	Workload (W)	254 ± 25	256 ± 28	0.20	−3.97; 0.52	0.310	<0.001	0.986	1.72	−8.67; 12.11	1.97	1.08	3.75	10.39
	HR (beats·min^−1^)	146 ± 10	145 ± 8	0.66	−1.66; 2.79	0.102	<0.001	0.902	−0.56	−10.86; 9.73	1.10	1.10	3.72	10.30
Dmax_LE_	Workload (W)	275 ± 27	280 ± 31	0.02 ^§^	−9.46; −1.99	0.618	<0.001	0.972	5.73	−11.56; 23.01	4.11	1.96	6.24	17.29
	HR (beats·min^−1^)	153 ± 10	154 ± 8	0.52	−3.79; 1.71	0.153	<0.001	0.849	1.04	−11.68; 13.76	1.85	1.66	4.59	12.72
AT_86HRmax_	Workload (W)	267 ± 32	273 ± 36	0.13	−13.20; 0.66	0.365	<0.001	0.954	6.27	−25.78; 38.31	5.20 *	2.94 *	11.56	32.05
	HR (beats·min^−1^)	151 ± 7	152 ± 7	0.09	−2.18; −0.45	0.420	<0.001	0.949	1.11	−3.83; 6.05	1.45	0.94	1.78	4.94
LT_3.5mM_	Workload (W)	261 ± 36	265 ± 34	0.29	−10.01; 2.30	0.253	<0.001	0.965	3.86	−24.65; 32.36	4.68 *	3.08 *	10.28	28.50
	HR (beats·min^−1^)	148 ± 14	148 ± 9	0.99	−3.62; 3.69	0.004	<0.001	0.798	−0.04	−16.95; 16.87	3.44 *^$^	3.14 ^$^*	6.10	16.91
LT2	Workload (W)	240 ± 30	242 ± 32	0.57	−9.01; 4.55	0.132	<0.001	0.958	2.23	−29.17; 33.62	3.42	3.14 *	11.32	31.39
	HR (beats·min^−1^)	141 ± 12	141 ± 9	0.65	−2.25; 3.90	0.108	<0.001	0.812	−0.82	−15.03; 13.38	2.80 *	2.41 ^$^*	5.13	14.21
LT_4mM_	Workload (W)	271 ± 39	276 ± 36	0.29	−10.80; 2.42	0.256	<0.001	0.832	4.19	−26.39; 34.77	5.44 *	3.19 *	11.03	30.58
	HR (beats·min^−1^)	152 ± 14	152 ± 9	0.95	−4.02; 3.75	0.014	<0.001	0.793	0.13	−17.87; 18.14	3.61 *^$^^†^	3.24 ^$^*^†^	6.49	18.00
LE_+1.5mM_	Workload (W)	240 ± 32	242 ± 32	0.46	−8.97; 3.52	0.176	<0.001	0.962	2.72	−26.19; 31.63	5.11 *	3.44 *^†^	10.42	28.91
	HR (beats·min^−1^)	141 ± 12	141 ± 10	0.69	−2.36; 3.81	0.094	<0.001	0.833	−0.72	−15.01; 13.57	2.60 *	2.22 ^$^*	5.15	14.29
Dmax_0.4_	Workload (W)	277 ± 35	279 ± 34	0.62	−10.17; 5.59	0.117	<0.001	0.954	2.29	−34.18; 38.76	7.28 *	3.70 *	13.16	36.47
	HR (beats·min^−1^)	153 ± 13	154 ± 9	0.87	−4.06; 3.37	0.037	<0.001	0.786	0.34	−16.86; 17.55	3.39 *^$^^†^	2.80 ^$^*^†^	6.21	17.20
LT1_+1.5mM_	Workload (W)	235 ± 37	238 ± 48	0.47	−11.76; 4.80	0.169	<0.001	0.945	3.48	−34.86; 41.82	4.95 *	4.03 *^†^	13.83	38.34
	HR (beats·min^−1^)	140 ± 12	140 ± 10	0.84	−2.59; 3.30	0.049	<0.001	0.810	−0.36	−14.00; 13.29	2.56 *^$^^†^	2.70 ^$^*	4.92	13.65

**SD**, standard deviation; **ICC**, intraclass correlation coefficient; **CV**, coefficient of variation; **SEM**, standard error of measurement; **MDC**, minimum detectable change; **MMCT**, maximal multistage cycling test; **[La^−^]**, blood lactate concentration; **CI**, confidence intervals; **ES**, effect size; **LoA**, limits of agreement; **AT_73Pmax_**, workload at 73% of **Pmax**; Dmax_LE_, workload at the maximum perpendicular distance from the straight line between the [La^−^] associated with the Minimum Lactate Equivalent and final [La^−^]; **AT_86HRmax_**, workload at 86% of maximal heart rate; **LT_3.5mM_** and **LT_4mM_**, workloads associated with [La^−^] of 3.5 mmol·L^−1^ and 4 mmol·L^−1^, respectively; **LT2**, workload at 1.5 mmol·L^−1^ above the average of the first four [La^−^] values of exercise; **LE_+1.5mM_**, workload at Minimum Lactate Equivalent plus 1.5 mmol·L^−1^. **Dmax_0.4_**, workload at the maximum perpendicular distance from the straight line between the [La^−^] associated with the previous stage to the one that the [La^−^] increased ≥0.4 mmol·L^−1^ and final [La^−^]. **LT1_+1.5mM_**, the workload at 1.5 mmol·L^−1^ above the [La^−^] associated with the lowest stage above, which [La^−^] increased by ≥0.1 mmol·L^−1^ in the following stage and ≥0.2 mmol·L^−1^ in the subsequent stage. **^§^** Significant difference between the two MMCT; * Significantly different from AT_73Pmax_; ^†^ Significantly different from Dmax_LE_; **^$^** Significantly different from AT_86HRmax_ (*p* ≤ 0.05). The symbols show differences with respect to the way the anaerobic threshold is specifically expressed, HR or Workload.

**Table 2:** Validity of workloads at anaerobic thresholds measured through MMCT for MLSS_W_ estimation (*n* = 16).

	Mean Differences	Pearson	Bland Altman (W)		SEE (W)
	*p*	r	Bias (Mean ± SD)	LoA	
AT_73Pmax_	0.001	0.94	−16.6 ± 16.8	−49.5; 16.2	16.8
Dmax_LE_	0.295	0.95	4.7 ± 16.2	−27.1; 36.4	15.7
AT_86HRmax_	0.491	0.93	−2.9 ± 16.2	−34.6; 28.9	18.3
LT_3.5mM_	0.117	0.93	−9.2 ± 22.1	−52.4; 34.0	18.0
LT2	<0.001	0.94	−31.4 ± 17.3	−65.3; 2.4	16.4
LT_4mM_	0.835	0.93	1.3 ± 24	−46.0; 48.6	18.7
LE_+1.5mM_	<0.001	0.91	−31.0 ± 23	−76.1; 14.1	20.7
Dmax_0.4_	0.222	0.95	5.4 ± 17.0	−28.0; 38.8	15.9
LT1_+1.5mM_	<0.001	0.88	−38.0 ± 25.6	−88.2; 12.3	23.2

**Table 3:** Correction equations for LT1_+1.5mM_, LE_+1.5mM_, LT2, LT_3.5mM_, and AT_73Pmax_.

	Correction Equation	Bland Altman (W)	
		Bias (Mean ± SD)	LoA
LT1_+1.5mM_	Corrected LT1_+1.5mM_ = 33.76 + (0.999 ∗ LT1_+1.5mM_)	−4.4 ± 25.60	−54.61; 45.74
LE_+1.5mM_	Corrected LE_+1.5mM_ = 6.643+(1.089 ∗ LE_+1.5mM_)	−3.22 ± 24.23	−50.71; 44.28
LT2	Corrected LT2 = 4.794+ (1.110 ∗ LT2)	−0.56 ± 18.11	−36.06; 34.95
LT_3.5mM_	Corrected LT_3.5mM_ = 34.088+ (0.894 ∗ LT_3.5mM_)	−2.56 ± 20.49	−42.71; 37.60
AT_73Pmax_	Corrected AT_73Pmax_ = −69.893 + (1.332 ∗ AT_73Pmax_)	−2.98 ± 18.56	−39.36; 33.39

**SD**, standard deviation; **LoA**, limits of agreement; **AT_73Pmax_**, workload at 73% of Pmax; **LT_3.5mM_**, workload associated with [La^−^] of 3.5 mmol·L^−1^; **LT2**, workload at 1.5 mmol·L^−1^ above the average of the first four [La^−^] values of exercise; **LE_+1.5mM_**, workload at Minimum Lactate Equivalent plus 1.5 mmol·L^−1^. **LT1_+1.5mM_**, the workload at 1.5 mmol·L^−1^ above the [La^−^] associated with the lowest stage above, which [La^−^] increased by ≥0.1 mmol·L^−1^ in the following stage and ≥0.2 mmol·L^−1^ in the subsequent stage.

## Data Availability

The data generated and analyzed during this study are available from the corresponding author upon reasonable request.

## Abbreviations

ANOVA	Analysis of variance
AT	Anaerobic threshold
AT_73Pmax_	The workload corresponding to 73% of the maximal aerobic workout
AT_86HRmax_	The workload associated with the 86% of HRmax
CI	Confidence intervals
CLT	Constant load tests
CV	Coefficient of variation
Dmax_0.4_	The workload at the maximum perpendicular distance from the straight line between the blood lactate concentration associated with the previous stage to the one that the blood lactate concentration increased ≥0.4mmol·L^−1^ and final blood lactate concentration data point in the third-order polynomial curve describing the blood lactate concentration kinetics during the maximal multistage cycling test
Dmax_LE_	The workload at the maximum perpendicular distance from the straight line between the blood lactate concentration associated with the minimum lactate equivalent and final blood lactate concentration data point in the third-order polynomial curve describing the blood lactate concentration kinetics during the maximal multistage cycling test
ES	Effect size
HRmax	Maximal heart rate
ICC	Intraclass correlation coefficient
[La^−^]	Blood lactate concentration
LE_min_	Minimum lactate equivalent
LE_+1.5mM_	The workload corresponding to the minimum lactate equivalent plus 1.5 mmol·L^−1^
LoA	Limits of agreement method
LT1_+1.5mM_	The workload at 1.5 mmol·L^−1^ above the blood lactate concentration associated with the lowest stage above which blood lactate concentration increased by ≥0.1 mmol·L^−1^ in the following stage and ≥0.2 mmol·L^−1^ in the subsequent stage
LT2	The workload at 1.5 mmol·L^−1^ in the individual blood lactate concentration vs. workload second-order polynomial curves above the average of the first four blood lactate concentrations values of exercise
LT_3.5mM_	The workloads associated with fixed blood lactate concentration of 3.5 mmol·L^−1^
LT_4mM_	The workloads associated with fixed blood lactate concentration of 4 mmol·L^−1^
MDC	Minimum detectable change
MLSS_W_	Exercise intensity corresponding to the maximal lactate steady state
MMCT	Maximal multistage cycling test
Pmax	Maximal aerobic workout
RPE	Rating of perceived exertion
SD	Standard deviation
SEE	Standard error of the estimate
SEM	Standard error of measurement
